# The Imidazoquinoline Toll-Like Receptor-7/8 Agonist Hybrid-2 Potently Induces Cytokine Production by Human Newborn and Adult Leukocytes

**DOI:** 10.1371/journal.pone.0134640

**Published:** 2015-08-14

**Authors:** Lakshmi Ganapathi, Simon Van Haren, David J. Dowling, Ilana Bergelson, Nikunj M. Shukla, Subbalakshmi S. Malladi, Rajalakshmi Balakrishna, Hiromi Tanji, Umeharu Ohto, Toshiyuki Shimizu, Sunil A. David, Ofer Levy

**Affiliations:** 1 Division of Infectious Diseases, Boston Children’s Hospital, Boston, MA, United States of America; 2 Harvard Medical School, Boston, MA, United States of America; 3 Department of Medicinal Chemistry, University of Kansas, Lawrence, KS, United States of America; 4 Graduate School of Pharmaceutical Sciences, University of Tokyo, Tokyo, Japan; Imperial College London, UNITED KINGDOM

## Abstract

**Background:**

Newborns and young infants are at higher risk for infections than adults, and manifest suboptimal vaccine responses, motivating a search for novel immunomodulators and/or vaccine adjuvants effective in early life. In contrast to most TLR agonists (TLRA), TLR8 agonists such as imidazoquinolines (IMQs) induce adult-level Th1-polarizing cytokine production from human neonatal cord blood monocytes and are candidate early life adjuvants. We assessed whether TLR8-activating IMQ congeners may differ in potency and efficacy in inducing neonatal cytokine production *in vitro*, comparing the novel TLR7/8-activating IMQ analogues Hybrid-2, Meta-amine, and Para-amine to the benchmark IMQ resiquimod (R848).

**Methods:**

TLRA-induced NF-κB activation was measured in TLR-transfected HEK cells. Cytokine production in human newborn cord and adult peripheral blood and in monocyte-derived dendritic cell cultures were measured by ELISA and multiplex assays. X-ray crystallography characterized the interaction of human TLR8 with Hybrid-2.

**Results:**

Hybrid-2 selectively activated both TLR7 and 8 and was more potent than R848 in inducing adult-like levels of TNF-α, and IL-1β. Consistent with its relatively high *in vitro* activity, crystallographic studies suggest that absence in Hybrid-2 of an ether oxygen of the C2-ethoxymethyl substituent, which can engage in unfavorable electrostatic and/or dipolar interactions with the carbonyl oxygen of Gly572 in human TLR8, may confer greater efficacy and potency compared to R848.

**Conclusions:**

Hybrid-2 is a selective and potent TLR7/8 agonist that is a candidate adjuvant for early life immunization.

## Introduction

There are more than 2,000,000 deaths per year worldwide due to infection in those less than 6 months of age [1–3]. The increased vulnerability of newborns and infants to infections as compared to older children and adults has been attributed to distinct early life immunity [4]. Given the significant morbidity and mortality associated with infections in early life, there is a pressing need for safe immunization early in life. However, distinct immunity in early life poses a challenge for developing neonatal and infant vaccines [5]. Vaccine efficacy in those with weakened immunity at the extremes of age, may require addition of an adjuvant to instruct and enhance the immune response [6]. Live attenuated or killed whole-cell vaccines provide intrinsic adjuvant activity, whereas subunit vaccines are adjuvanted with Alum, oil-in-water emulsions such as MF-59 or purified microbial products such as monophosphoryl lipid A (MPLA; *Toll*-like-receptor (TLR) 4 agonist (TLR4A). However, adjuvants have age-specific immune modulating effects such that not all adjuvants are effective in early life [7].

Neonatal and infant immunity is functionally distinct from that of adults due to both cellular and soluble immunosuppressive factors [8,9] and is biased towards the induction of regulatory T cell (T_reg_) or T-helper type 2 (Th_2_) responses, which may limit immune responses to intracellular pathogens and to vaccines directed against them [10]. Insights into the mechanisms that limit early life immune responses have informed the search for and development of age-specific adjuvanted vaccine formulations [4,10–12]. One promising approach to enhance the immunity of newborns and infants is the use of TLRAs as either stand-alone immunomodulators to enhance innate immune defense against infection and/or as vaccine adjuvants [13–15] TLRAs, including single stranded ribonucleic acid (ssRNA: a TLR8 agonist), are present in live attenuated vaccines such as the Japanese Encephalitis Vaccine and Yellow Fever Vaccine 17D (YF-17D) that activate multiple dendritic cell (DC) subsets via TLR2, TLR7, TLR8, and TLR9 [16,17].

Newborn whole blood, cord blood mononuclear cells and monocytes demonstrate impaired production of the pro-inflammatory, Th1-polarizing cytokine Tumor Necrosis Factor (TNF) to TLR 1-7As [12]. In contrast, TLR8As may possess unique activity in newborns [18,19]. TLR7, -8 and -9 are activated by nucleic acids, and are located in the endosomal compartment [18–20]. Human dermal and myeloid DCs, as well as T cells, express TLR8 [21]. TLR8As, including dual TLR 7/8As, such as those of the small, synthetic imidazoquinoline (IMQ) family of anti-viral molecules activate robust immune responses in newborn cells as these agents are refractory to endogenous suppressive factors, such as adenosine, present in newborn blood plasma [22]. For example, R848 a TLR-7/8A induces adult levels of TNF and interleukin (IL)-12p40/70 production in newborn whole blood and cord blood mononuclear cells (CBMCs) [19]. Although agonists of the same TLR can vary substantially in their relative potency [23], little is known regarding the relative activity of different TLR8As towards human leukocytes.

A family of novel, low molecular weight synthetic IMQs that selectively activate cells via TLR7 and/or -8 are being evaluated as vaccine adjuvants [24,25]. A key approach to enhance adjuvant potency is the use of medicinal chemistry to create congeners with more favorable properties [26,27]. In this study, we characterized three TLR7/8 dual-agonistic compounds for Th1- and Th17-polarizing cytokine induction in human newborn and adult leukocytes. We found that Hybrid-2, a TLR7/8 selective imidazoquinoline agonist, demonstrates greater cytokine-inducing potency in both newborn and adult leukocytes compared to R848. Crystallographic studies suggest that absence in Hybrid-2 of an ether oxygen of the C2-ethoxymethyl substituent present in R848 that engages in unfavorable electrostatic and/or dipolar interactions with the carbonyl oxygen of Gly572 in human TLR8 may contribute to its greater potency compared to R848. Thus, Hybrid-2 may be a particularly attractive immunomodulator and adjuvant candidate for early life immunization.

## Materials and Methods

### Ethics Statement

Non-identifiable cord blood samples were taken with approval from the Ethics Committee and Institutional Review Boards of The Brigham and Women’s Hospital, Boston, MA and Beth Israel Deaconess Medical Center, Boston, MA. Adult blood samples were collected from volunteer donors following written informed consent with approval from the Ethics Committee and the Institutional Review Board of Boston Children’s Hospital, Boston, MA. Human experimentation guidelines of the U.S. Department of Health and Human Services, The Brigham and Women’s Hospital, Beth Israel Deaconess Medical Center and Boston Children’s Hospital were observed.

### TLR Agonists and Assay Reagents

The following IMQ TLRAs were synthesized from diaminoquinoline analogues at the University of Kansas, KS: Para-amine (TLR7/8), Meta-amine (TLR7/8), Hybrid-2 (TLR7/8) as previously described [24]. The commercially available TLR7/8 agonist, R848 (InvivoGen, San Diego, CA) was also used at the concentration noted in the figure legends. TLRAs were verified to be free of endotoxin (< 1 EU/ml) as measured by the *Limulus amoebocyte lysate* (LAL) assay per the manufacturer’s instructions (Charles River, Wilmington, MA). TLRAs were prepared in dimethyl sulfoxide (DMSO) (Sigma-Aldrich, St Louis, MO).

### Human TLR expressing HEK 293 Cell Assays

Agonist specificity was characterized using TLR-transfected human embryonic kidney (HEK) cells. Briefly, the induction of nuclear factor (NF)-κB was quantified using human TLR2-5 and 7-9-specific HEK-Blue (InvivoGen) reporter gene assays as previously described [24,25]. HEK293 cells stably co-transfected with the appropriate human TLR (hTLR), and secreted alkaline phosphatase (sAP), were maintained in HEK-Blue Selection medium containing zeocin and normocin. Stable expression of sAP under control of NF-κB/AP-1 promoters is inducible by cognate TLR agonists, and extracellular sAP in the supernatant is proportional to NF-κB induction. HEK-Blue cells were incubated at a density of ~10^5^ cells/ml in a volume of 80 μl/well, in 384-well, flat-bottomed, cell culture-treated microtiter plates until confluency was achieved, and subsequently stimulated with graded concentrations of stimuli. sAP was assayed spectrophotometrically using an alkaline phosphatase-specific chromogen (present in HEK-detection medium as supplied by the vendor) at 620 nm.

### Human TLR8 expression, purification and crystallization

The extracellular domain of hTLR8 (residues 27–827) was prepared as described previously [28] and was concentrated to 16 mg/ml in 10 mM 2-(*N*-morpholino)ethanesulfonic acid (MES) buffer (pH 5.5), 50 mM sodium chloride (NaCl). The protein solutions for the crystallization of hTLR8/compound complexes contained hTLR8 (8.5 mg/ml) and compound (protein:compound molar ratio of 1:10) in a crystallization buffer containing 7 mM MES (pH 5.5), 35 mM NaCl. Crystallization experiments were performed with sitting-drop vapor-diffusion methods at 293 K. Crystals of hTLR8/compound were obtained with reservoir solutions containing 9–12% (w/v) polyethylene glycol (PEG) 3350, 0.3 M potassium formate, and 0.1 M sodium citrate (pH 4.8–5.2).

### Crystallography data collection and structure determination

Diffraction datasets were collected on X-ray Beamlines PF-AR NE3A (Structural Biology Research Center, Ibaraki, Japan) and the SPring-8 BL41XU (Japan Synchrotron Radiation Research Institute (JASRI), Hyogo, Japan) under cryogenic conditions at 100 kelvin. Crystals of hTLR8/compound were soaked into a cryoprotectant solution containing 15% (w/v) PEG3350, 0.23 M potassium formate, 75 mM sodium citrate pH 4.8–5.2, 7.5 mM MES pH 5.5, 38 mM NaCl, and 25% glycerol. Datasets were processed using the HKL2000 package [29] or imosflm [30]. HTLR8/compound structures were determined by the molecular replacement method using the Molrep program [31] with the hTLR8/CL097 structure (Protein Data Bank (PDB) ID: 3W3J) as a search model. The model was further refined with stepwise cycles of manual model building using the COOT program [32] and restrained refinement using REFMAC [33] until the R factor was converged. Compound molecule, *N*-glycans, and water molecules were modeled into the electron density maps at the latter cycles of the refinement. The stereochemical quality of the final protein structure was evaluated with PROCHECK software [34]. The statistics of the data collection and refinement are also summarized in S2 Fig. The visualization software PyMOL was employed to generate representations of the determined crystal structures [35]. Coordinates for Hybrid-2 were deposited in the PDB of the Research Collaboratory for Structural Bioinformatics: PDB code: 4R6A.

### Human Blood

Human peripheral blood was collected from healthy adult volunteers, while human newborn cord blood was collected immediately after Cesarean section delivery of the placenta. Births to HIV-positive mothers were excluded. Human blood was anti-coagulated with 15–20 units/ml pyrogen-free sodium heparin (American Pharmaceutical Partners, Inc., Schaumberg, IL). All blood products were kept at room temperature and processed within 4 h of collection.

### Blood Sample Processing and *in vitro* Stimulation

Assessment of TLRA activity in whole blood was completed as previously described [36]. Briefly, neonatal cord blood or adult whole blood (WB) was mixed 1:1 with sterile pre-warmed (37°C) Rosewell Park Memorial Institute RPMI 1640 medium (Invitrogen, Carlsbad, CA) and 180 μL of the 1:1 suspension was added to each well of a 96 well U-bottom plate (Becton Dickinson, Franklin Lakes, NJ, USA) containing 20 μl freshly prepared TLR agonists at 10 x the final concentration. Vehicle (DMSO) was added separately as a control. Suspensions containing 200 μl/well were gently mixed by pipetting and incubating for 6 h at 37°C in a humidified incubator at 5% CO_2_. After culture, plates were centrifuged at 500 x g and 100–150 μl of supernatant was carefully removed by pipetting without disturbing the cell pellet. Supernatants derived from human leukocyte stimulations were assayed by enzyme-linked immunoabsorbent assay (ELISA) for TNF (BD Biosciences, San Jose, CA, USA) and IL-1β (eBiosciences, San Diego, CA). Experiments were performed in triplicates.

### Monocyte-derived Dendritic Cells (MoDCs)

Heparinized human newborn cord blood or adult peripheral blood was layered onto Ficoll-Hypaque (Ficoll-Paque PREMIUM, GE Healthcare, Waukesha, WI) and centrifuged for 30 minutes at 1000 x g to collect cord blood mononuclear cell layers (CBMC) or peripheral blood mononuclear cell layers (PBMC), respectively. Monocytes were isolated from mononuclear cell fractions by positive selection by magnetic microbeads according to the manufacturer’s instructions (Miltenyi Biotec, Auburn, CA) using CD14 as a pan marker for monocytes. Monocyte preparations were routinely assessed by flow cytometry for cluster of differentiation (CD) 14 expression and were routinely observed to be > 95%. Isolated monocytes were cultured in tissue culture dishes at 10^6^ cells/ml in RPMI 1640 media containing fresh 10% autologous plasma, supplemented with recombinant human (rh)IL-4 (50 ng/ml) and rhGM-CSF (100 ng/ml) (R&D Systems; Minneapolis, MN) with one additional supplement of fresh media and cytokines at day 3 of culture. After 6 days, immature MoDCs of > 90% purity (HLA-DR^+^, CD14^-^, DC-SIGN^+^ as ascertained by flow cytometry) were harvested by gently pipetting only the loosely adherent fraction and re-plated (10^5^ cells/well) in 96-well U-bottom plates in the presence or absence of TLRAs at the indicated concentrations.

### Flow Cytometry

Monocytes or MoDCs were resuspended in staining buffer (PBS, 0.5% human serum albumin (HSA)) and stained for 30 min at 4°C in the dark (1.5*10^5^/per staining) with fluorophore-labeled antibodies (HLA-DR-FITC and CD14-V450 or DC-SIGN-PE) (BD Biosciences). Cells were then centrifuged 500 x g for 10 mins, washed with PBS, fixed (1% paraformaldehyde (PFA)) and filtered prior to flow cytometry acquisition using an LSRFortessa cytometer (BD Biosciences) and analyzed using FlowJo software (TreeStar Inc., Ashland, OR).

### Statistical Analyses and Graphics

Analysis of data was performed using Prism for MacIntosh v. 5.0b (GraphPad Software Inc., San Diego, CA). Data in figures represent means ± SEM. For normal sample sets, two tailed t test was performed. Non-normal sample tests were analyzed by Mann-Whitney test, as appropriate. p values < 0.05 were considered statistically significant.

## Results

### Specificity of Imidazoquinoline Agonists for Human TLR7 and TLR8

**Fig 1:**
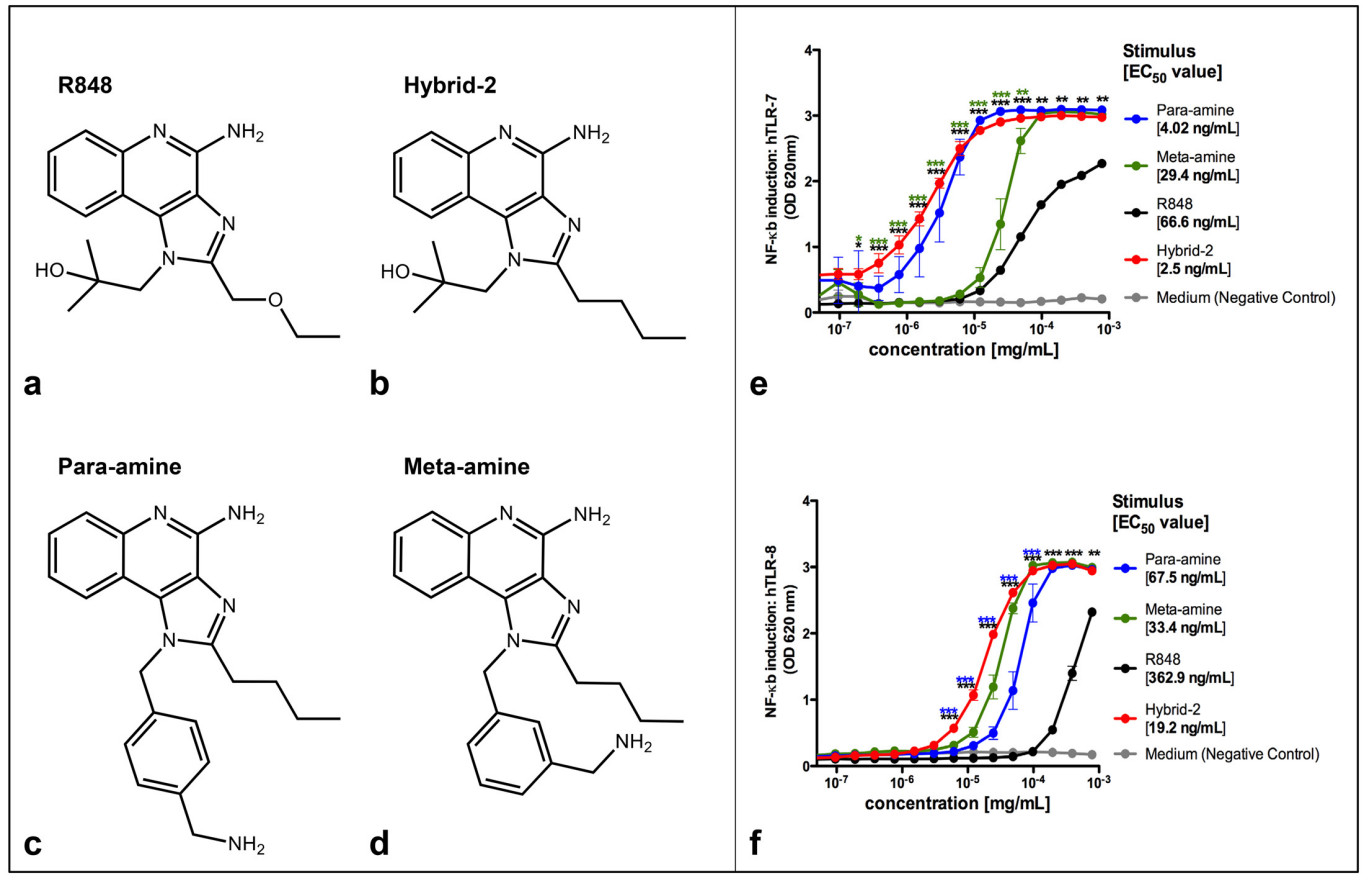
NF-κB induction by TLR7/8 agonists in HEK-TLR cells. Four TLR7/8 agonists were compared. (A) Structure of R848, (B) Structure of Hybrid-2, (C) Structure of para-amine and (D) Structure of meta-amine. HEK-293 cells transfected with (E) human TLR7 and (F) TLR8 and an NF-κB-driven reporter SEAP gene were stimulated for 18–24 h with TLR agonists. The y-axis shows the level of SEAP activity in the Quanti-blue assay optical density (OD). The x-axis shows the concentration of each compound in mg/ml. Each data point represents the mean ± SD of OD at 650 nm of triplicate culture wells. HEK detection medium alone (negative control) is represented in gray. The TLR7/8 benchmark agonist R848 is represented in black; The TLR 7/8 agonists Para-amine, Meta-amine and Hybrid-2 are represented in blue, green and red respectively. Three stars indicate significance of p < 0.001 (t test) and two green indicate significance of p < 0.01 (t test).

To assess the selectivity of the novel IMQ compounds (Fig 1a, 1b, 1c and 1d), we characterized NF-κB induction in HEK-293 cells selectively expressing human TLR7 or TLR8 (Fig 1e and 1f). Hybrid-2, Para-amine and Meta-amine demonstrated concentration-dependent activation of NF-κB in TLR7 and TLR8 transfected cells. We compared compound potency based on half maximal effective concentration (EC_50_), the concentration of agonist at which half maximal activation of NF-κB was observed in TLR7 and TLR8 transfected cells (Fig 1e and 1f). This analysis indicated the following rank of potencies for TLR7: Hybrid-2 (EC_50_ 2.5 ng/ml) > para-amine (EC_50_ 4.02 ng/ml) > meta-amine (EC_50_ 29.4 ng/ml) > R848 (estimated EC_50_ 66.6 ng/ml) and for TLR8: Hybrid-2 (EC_50_ 19.2 ng/ml) > meta-amine (EC_50_ 33.4 ng/ml) > para-amine (EC_50_ 67.5 ng/ml) > R848 (estimated EC_50_ 362.9 ng/ml). The specificity of Hybrid-2 was evaluated by demonstrating lack of ability to activate NF-κB in other HEK-cell lines transfected with TLR2,3,4,5 and 9 (S1 Fig).

### Hybrid-2 Potently Activates Human Newborn and Adult Leukocytes

**Fig 2:**
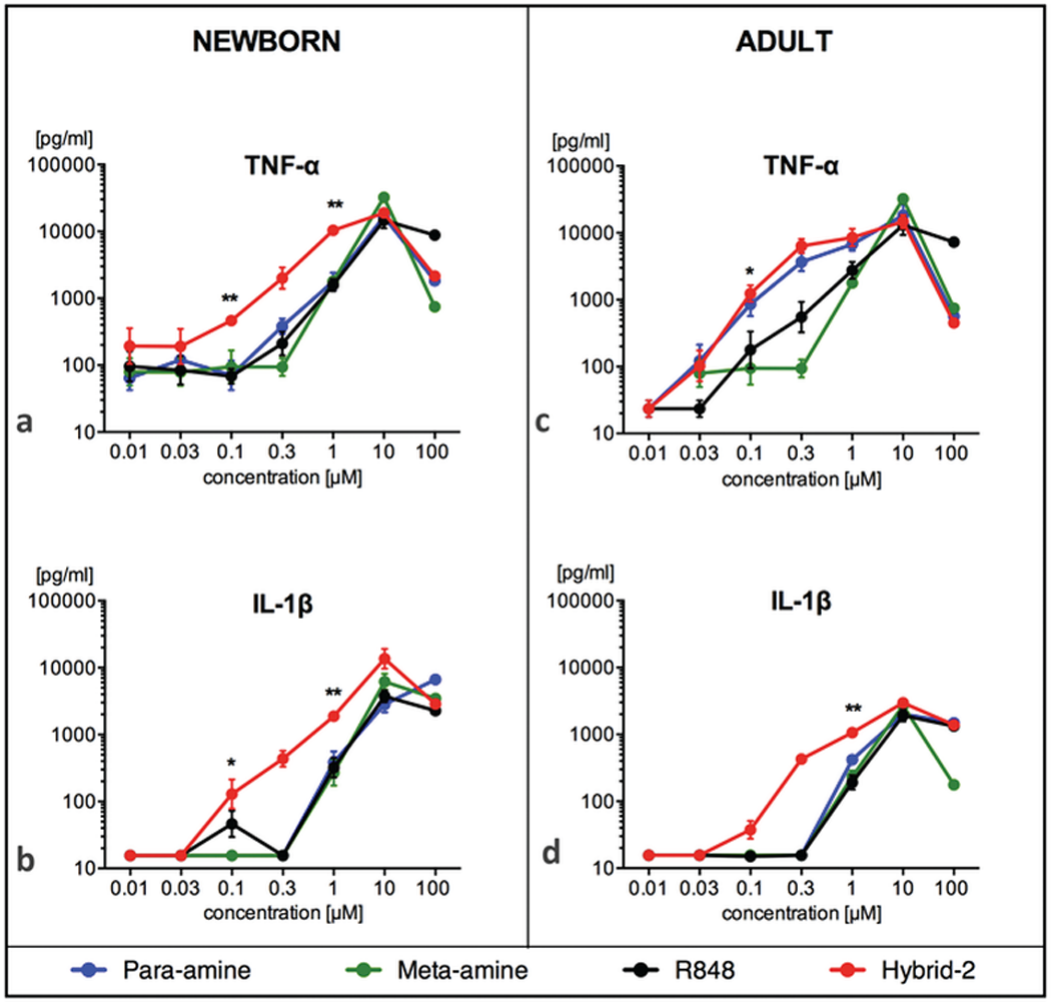
Hybrid-2 is more potent and effective than R848, para-amine and meta-amine in a whole blood cytokine assay. Human newborn and adult blood was cultured for 6h with TLR 7/8 agonists R848, para-amine, meta-amine and Hybrid-2 and supernatants collected for TNF or IL-1β ELISA. Compound concentrations are shown in μM. Data are shown as mean ± SEM of n = 6–8. For between-agonist analyses, t test was applied to compare Hybrid-2 to the other compounds. For between age-group analyses, t test was applied to compare Hybrid-2 and R848 in newborns and adults. Statistical significance is denoted as follows: *p<0.05 and **p<0.01, with black star(s) for comparison of Hybrid-2 to R848.

We tested the ability of the IMQ TLRAs to induce concentration-dependent cytokine production in human newborn and adult blood (Fig 2). All tested IMQs induced the production of TNF and IL-1β in a dose-dependent manner in both newborn cord blood and adult blood, significantly above baseline. As Hybrid-2 was the most potent of all four IMQ agonists, we tested the concentration-dependent cytokine induction of Hybrid-2 compared with R848 (Resiquimod), a TLR7/8A that has been studied as an adjuvant in non-human primates [37] and as a topical antiviral agent human clinical trials [38]. When compared to R848 at 0.1 μM and 1 μM, Hybrid-2 demonstrated greater efficacy in inducing TNF and IL-1β production in neonatal blood (Fig 2a and 2b N = 6, t test, p < 0.05). Hybrid-2 also demonstrated greater efficacy in stimulating TNF and IL-1β production in adult blood at 0.1 μM and 1 μM respectively (Fig 2c and 2d N = 6, t test, p < 0.05). Overall, Hybrid-2 demonstrated greatest potency (i.e. lowest EC_50_) of the agonists tested for both TNF and IL-1β production in newborn cord and adult peripheral blood (Fig 2, S1 Table). With respect to efficacy (magnitude) of cytokine induction, maximal cytokine responses were achieved at higher agonist concentrations of Hybrid-2 (e.g., 10 μM), similar to those induced by R848, Meta-amine, and Para-amine (Fig 2, S1 Table).

### Hybrid-2 induced similar TNF and IL-1β Production in Newborn and Adult Blood

Previous work has demonstrated the unique efficacy of TLR8 agonists in activation of neonatal antigen presenting cells [19]. Consistent with these findings, we noted that both R848 and Hybrid-2 induced concentrations of TNF and IL-1β in newborn cord blood at least as great as those induced in adult blood (Fig 2).

### Hybrid-2 Potently Activates Human Neonatal MoDCs

**Fig 3:**
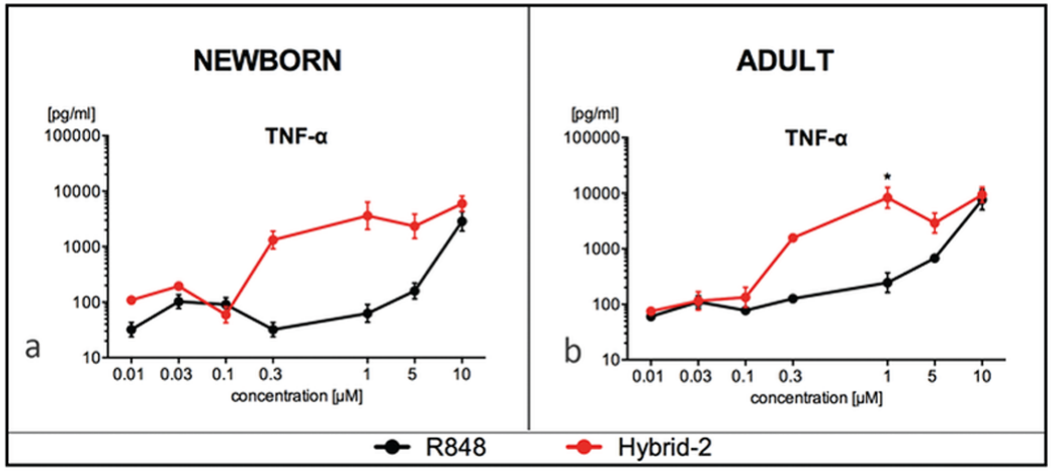
Hybrid-2 is more potent than R848 in inducing TNF production by human newborn and adult MoDCs. Stimulation of (A) newborn MoDCs and (B) adult MoDCs for 24 h with Hybrid-2 or R848. Supernatants collected for TNF ELISA. Compound concentrations are shown in μM. Data are shown as mean ± SEM of n = 5. For between-agonist analyses, t test was applied to compare Hybrid-2 to R848. Statistical significance is denoted as follows: *p<0.05 as a black star.

DCs are key antigen-presenting cells, important for the initiation of an immune response to adjuvanted vaccines [39]. To evaluate the effect of IMQs on DCs, we generated adult and neonatal monocyte-derived DCs (MoDCs). MoDCs were cultured in 10% autologous plasma, which we have previously established as a platform that allows for evaluation of age specific function of antigen presenting cells [18,23]. We compared concentration-dependent responses of human neonatal MoDCs to R848 and Hybrid-2 (both 0.1–10 μM) with respect to production of TNF. Both R848 and Hybrid-2 induced TNF production in a concentration-dependent manner as compared to unstimulated DCs. Hybrid-2 was more potent than R848 in inducing TNF, doing so at a concentration at ≥ 0.3 μM, compared to R848, which induced comparable levels of TNF only between 1–10 μM (Fig 3). In adult MoDCs, Hybrid-2 demonstrated greater TNF production compared to R848 at concentration of 1M (Fig 3b, N = 5, t test, p < 0.05). In newborn MoDCs, Hybrid-2 demonstrated greater TNF production compared to R848 at concentration of 0.3 M and 1 M with this difference approaching statistical significance (N = 5, t test, p = 0.06).

### Crystal structure of Hybrid-2 bound to Human TLR8

**Fig 4:**
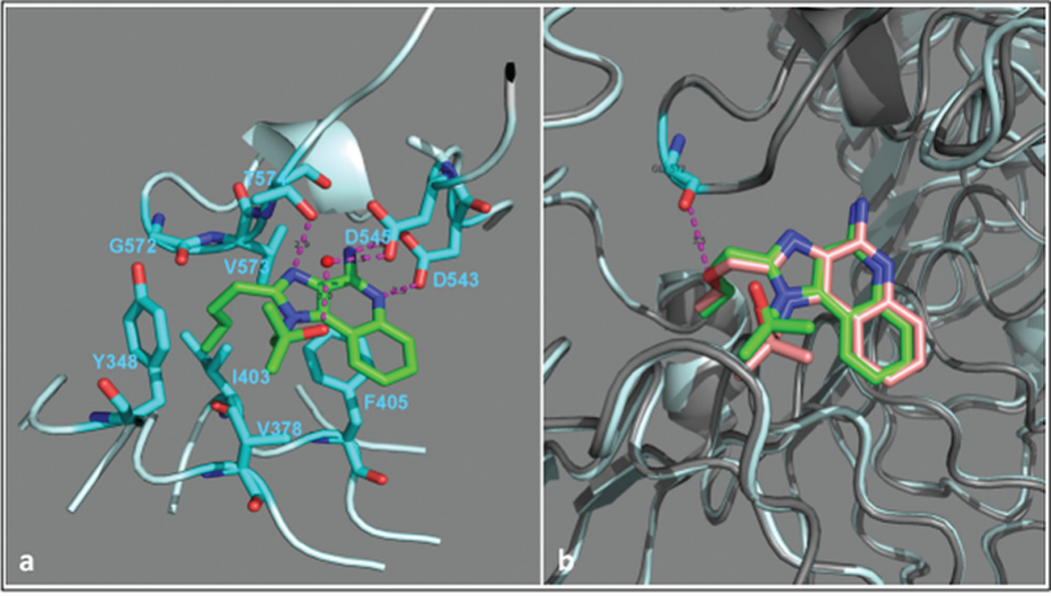
Crystal structure of Hybrid-2 and R848 with human TLR8. (A) The C-atoms of Hybrid-2 are shown in green, and the residues on TLR8 are shown in cyan. A water molecule involved in mediating the interaction between the hydroxyl on N^1^-substituent of Hybrid-2 and Asp545 residue is shown as a red sphere. The hydrogen bonds are represented as dashed lines in magenta, and the bond distances are marked in black. Overlay of crystal structures of Hybrid-2 and R848 with TLR8 is shown in (B). R848 is shown in light red and its TLR8 receptor shown in grey. The electrostatic repulsion between the backbone carbonyl of Gly572 residue and ether oxygen of the C2-ethoxymethyl substituent of R848 is shown in magenta.

To further characterize interactions between Hybrid-2 and human TLR8, we obtained a high-resolution (2.1 Å) structure of human TLR8 co-crystallized with Hybrid-2 (Fig 4a; the atomic coordinates and experimental data have been deposited in the Protein Data Bank under accession code 4R6A). An examination of the complex showed that the binding geometry and interactions for Hybrid-2 with TLR8 were similar to R848 [28]. The amidine group of Hybrid-2 demonstrated strong hydrogen bonding interactions with Asp543. Additionally, water-mediated hydrogen bonding interactions were observed between the hydroxyl on the *N* ^1^-2-methylpropan-2-ol substituent of Hybrid-2 and Asp545 residue. Other key interactions include hydrogen bonding of the *N* ^3^-amine of the imidazole ring with the backbone amide-NH of the Thr574 residue, π-π stacking interactions of the benzene ring of the quinoline moiety with the side chain of Phe405, and hydrophobic interactions of the aliphatic C2-*n*-butyl chain of Hybrid-2 in a pocket formed by the Tyr348, Val378, Ile403, Phe405, Gly572, and Val573 residues (Fig 4a). The only difference between the structures of Hybrid-2 and R848 is that Hybrid-2 lacks the ether oxygen on the C2-substituent (see structures in Fig 1). Accordingly, the lower potency of R848 may be attributable to unfavorable electrostatic and/or dipolar interactions between the ether oxygen of the C2-ethoxymethyl substituent and the carbonyl oxygen of the Gly572 residue (Fig 4b).

## Discussion

Activation of innate immune cells is a key component of effective vaccine responses [6]. Several current licensed vaccines achieve effective induction of these innate immune responses by either employing live attenuated organisms or microbial components with intrinsic TLRA activity [40]. However, live attenuated vaccines are contraindicated in sub-groups of patients including immunocompromised populations. Multiple sub-unit vaccines are safe and effective parts of the immunization schedule, but some, such as acellular pertussis vaccine, demonstrate sub-optimal activity suggesting that addition of an adjuvant may be beneficial [5]. Hence, characterizing the immunostimulatory properties of candidate adjuvants is important to evaluate their translational potential. In this context, we report for the first time the *in vitro* cytokine inducing activity of a potent and selective TLR7/8A, Hybrid-2 as well as its structural interactions with the human TLR8 receptor.

We demonstrate that Hybrid-2 potently targets TLR7/8 and propose that, like previously studied TLR7/8As, its Th-cytokine inducing activity is refractory to soluble and cellular suppressive mechanisms such as the neonatal adenosine system [18] and suppressive erythroid precursors [8,41]. Tested in both whole blood and MoDC assays, Hybrid-2 (EC_50_ 0.3 μM) was both more effective and potent than R848 (EC_50_ 1.0 μM). The potential benefits of increased efficacy and potency of adjuvants may include antigen dose sparing as well as reduction in the number of vaccine doses required.

Molecular modeling studies of TLR8As have suggested that binding affinity plays an important role in species-specificity of these agonists [42]. To gain insight into potential mechanism(s) underlying Hybrid-2’s potency we characterized the crystal structure of hTLR8:Hybrid-2. Transposing the N^1^ and C2 substituents on these IMQ molecules resulted in varying TNF inducing potencies, which may be due to differences in binding affinity to the TLR7 or TLR8. To further characterize interactions between Hybrid-2 and TLR8, we defined the crystal structure of hybrid-2 co-crystalized with human TLR8 and in comparison to R-848 (Fig 4). Both R848 and Hybrid-2 demonstrate strong hydrogen bonding with Asp543 of TLR8. Hybrid-2, however, lacks the ether oxygen on the C2-substituent. Unfavorable electrostatic and/or dipolar interactions between the ether oxygen of the C2-ethoxymethyl substituent of R848 and the carbonyl oxygen of the Gly572 residue of TLR8 may contribute to the lower potency of R848 as compared to Hybrid-2. These observations raise the possibility that that the higher potency of Hybrid-2 vs. R848 in inducing TNF and IL-1β production may be due to higher binding affinity of Hybrid-2 to TLR8.

To the extent that our *in vitro and ex-vivo* studies accurately model immune responses *in vivo*, our findings support the potential adjuvant activity of Hybrid-2 in early life as well as in adults. Future translational studies may include assessing the safety and efficacy of Hybrid-2-containing vaccine formulations in newborn animals, including non-human primates, that express TLR 7/8 functionally and structurally similar to humans [40]. Hybrid-2’s potency is such that lower adjuvant doses, relative to other IMQ analogs, may suffice in vaccine formulations thereby potentially reducing adjuvant- and antigen-dose as well as reactogenicity. The potency of Hybrid-2 may also be advantageous with respect to its potential development as a “stand-alone” TLRA that may serve as an immunomodulator to boost innate defense against infection [43], reduce atopy and allergy [44] as well as treat cancer [45].

In summary, we have identified the IMQ Hybrid-2 as a potent inducer of TNF and IL-1β by human newborn blood leukocytes and MoDCs. The relatively high potency and efficacy of Hybrid-2 may render it a candidate “stand-alone” immunomodulator and/or adjuvant for novel early life vaccines.

## Supporting Information

S1 Fig Hybrid-2 does not induce NF- *κB* in HEK-293 cells transfected with TLR2,3,4,5,9. HEK-293 cells transfected with (A) Human TLR2, (B) Human TLR3, (C) Human TLR4, (D) Human TLR5 and (E) Human TLR9 an NF-κB-driven reporter SEAP gene were stimulated for 18–24 h with Hybrid-2, positive control as indicated and detection medium (negative control). The y-axis shows the level of SEAP activity in the Quanti-blue assay optical density (OD). The x-axis shows the concentration of each compound in mg/ml. Each data point represents the mean ± SD of OD at 650 nm of triplicate culture wells. HEK detection medium alone (negative control) is represented in gray, positive control is represented in black and Hybrid-2 is represented in red.
(TIF)

S2 Fig Data collection and refinement of Hybrid-2. Values in parentheses are for the shell with the highest resolution. R_merge_(I) = Σ|I − <I>|/ΣI,where I is the diffraction intensity. R = Σ|F_o_ − F_c_|/ΣF_o_, where F_o_ and F_c_ are the observed and calculated structure amplitudes, respectively. R_free_ is an R value for a 5% subset of all reflections, but was not used in the refinement
(TIF)

S1 Table Tabular representation of data displayed in Fig 2. Human newborn and adult blood was cultured for 6h with TLR 7/8 agonists R848, para-amine, meta-amine and Hybrid-2 and supernatants collected for TNF or IL-1β ELISA. Compound concentrations are shown in μM/ml. Cytokine production is shown as pg/ml.
(PDF)
