# Supplementary figures and images for: The Imidazoquinoline Toll-Like Receptor-7/8 Agonist Hybrid-2 Potently Induces Cytokine Production by Human Newborn and Adult Leukocytes
Source: PLoS One. 2015 Aug 14;10(8):e0134640. doi: 10.1371/journal.pone.0134640 (PMC4537157; doi:10.1371/journal.pone.0134640)

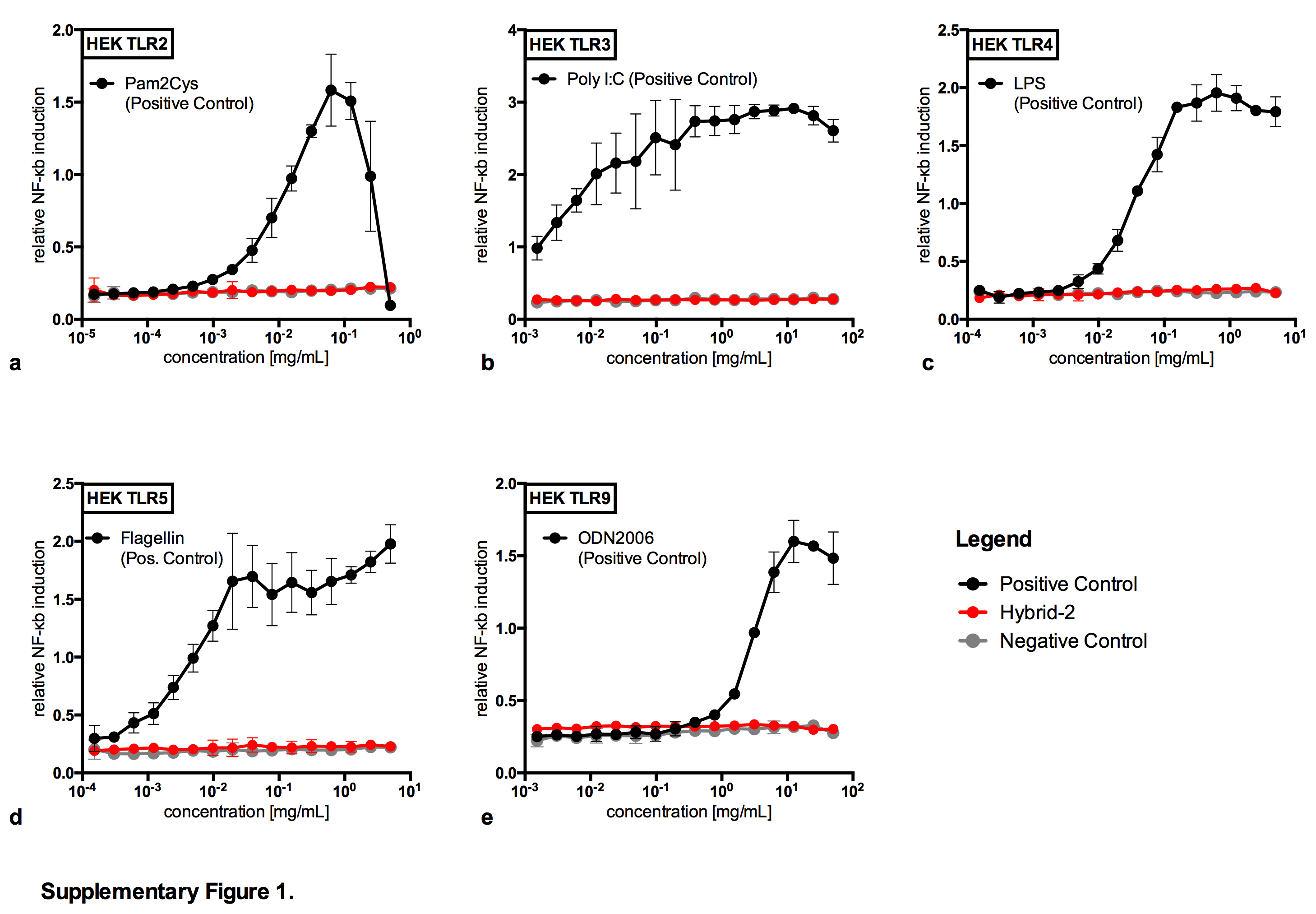

Supplement: S1 Fig — HEK-293 cells transfected with (A) Human TLR2, (B) Human TLR3, (C) Human TLR4, (D) Human TLR5 and (E) Human TLR9 an NF-κB-driven reporter SEAP gene were stimulated for 18–24 h with Hybrid-2, positive control as indicated and detection medium (negative control). The y-axis shows the level of SEAP activity in the Quanti-blue assay optical density (OD). The x-axis shows the concentration of each compound in mg/ml. Each data point represents the mean ± SD of OD at 650 nm of triplicate culture wells. HEK detection medium alone (negative control) is represented in gray, positive control is represented in black and Hybrid-2 is represented in red. (TIF) [file pone.0134640.s001.tif]

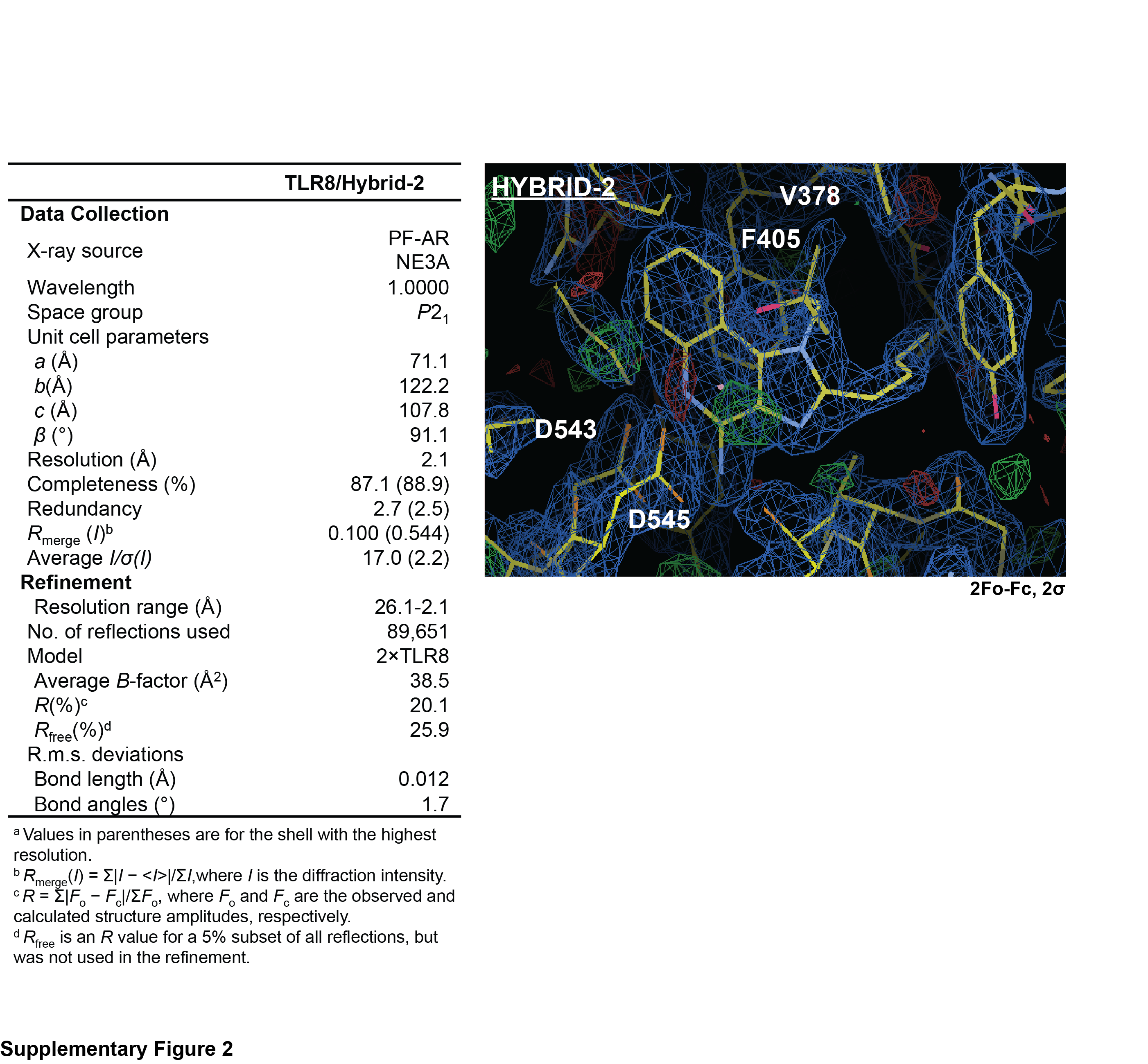

Supplement: S2 Fig — Values in parentheses are for the shell with the highest resolution. Rmerge(I) = Σ|I − |/ΣI,where I is the diffraction intensity. R = Σ|Fo − Fc|/ΣFo, where Fo and Fc are the observed and calculated structure amplitudes, respectively. Rfree is an R value for a 5% subset of all reflections, but was not used in the refinement (TIF) [file pone.0134640.s002.tif]
