# Supplementary material for: The Imidazoquinoline Toll-Like Receptor-7/8 Agonist Hybrid-2 Potently Induces Cytokine Production by Human Newborn and Adult Leukocytes
Source: PLoS One. 2015 Aug 14;10(8):e0134640. doi: 10.1371/journal.pone.0134640 (PMC4537157; doi:10.1371/journal.pone.0134640)
Supplement: S1 Table — Human newborn and adult blood was cultured for 6h with TLR 7/8 agonists R848, para-amine, meta-amine and Hybrid-2 and supernatants collected for TNF or IL-1β ELISA. Compound concentrations are shown in μM/ml. Cytokine production is shown as pg/ml. (PDF) [file pone.0134640.s003.pdf]

Supplementary Table S1

Hybrid 2 showed the lowest EC50 values (indicating greatest potency) of all agonists tested for both TNF and IL-1b production in adult peripheral blood. The maximum cytokine responses achieved at higher agonist concentrations of Hybrid 2 (e.g., 10mM) was similar to those induced by R848, Meta-amine, and Para-amine.

Hybrid-2

Concentration [μM]	Newborn		Adult	
	TNF-α	IL-1β	TNF-α	IL-1β
0.01	193.14	15.63	23.44	15.63
0.03	189.84	15.63	102.83	15.63
0.1	464.55	129.48	1427.29	37.42
0.3	1998.77	437.70	6362.58	427.95
1	3750.42	1169.67	14269.07	1061.65
10	10395.78	1887.36	20983.61	2972.60

Meta-amine

Concentration [μM]	Newborn		Adult	
	TNF-α	IL-1β	TNF-α	IL-1β
0.01	79.15	15.63	23.44	15.63
0.03	78.72	15.63	23.44	15.63
0.1	94.65	15.63	20.83	15.63
0.3	93.72	15.63	214.57	15.63
1	1779.40	271.87	3739.68	232.86
10	32214.43	6199.29	19087.54	2699.66

Para-amine

Concentration [μM]	Newborn		Adult	
	TNF-α	IL-1β	TNF-α	IL-1β
0.01	64.05	15.63	23.44	15.63
0.03	120.31	15.63	121.74	15.63
0.1	70.49	15.63	856.34	15.63
0.3	381.52	15.63	3653.12	15.63
1	1780.16	387.41	6740.50	419.70
10	16542.73	2820.52	18165.00	2008.09

R848

Concentration [μM]	Newborn		Adult	
	TNF-α	IL-1β	TNF-α	IL-1β
0.01	95.80	15.63	23.44	15.63
0.03	84.29	15.63	23.44	15.63
0.1	68.30	46.36	284.13	14.94
0.3	210.98	15.63	549.52	15.63
1	654.85	415.65	3939.41	191.49
10	1574.58	322.08	19454.73	1922.12
